# The Effects of Oregano Essential Oil on Intestinal Health and Muscle Nutritional Components of Crayfish (*Procambarus clarkii*)

**DOI:** 10.3390/ani16182926

**Published:** 2026-09-17

**Authors:** Zhipeng Huang, Juan Li, Jian Zhou, Yang Feng, Qiang Li, Han Zhao, Zhongmeng Zhao, Huadong Li, Xiaohong Liao, Yuanliang Duan, Lu Zhang

**Affiliations:** Fisheries Research Institute, Sichuan Academy of Agricultural Sciences (Sichuan Fisheries Research Institute), Chengdu 611731, China; h3392078@163.com (Z.H.); lijuansuibian@163.com (J.L.); zhoujian980@126.com (J.Z.); fyang@scsaas.cn (Y.F.); liq7920@126.com (Q.L.); zhaohan232323@163.com (H.Z.); 18227552594@163.com (Z.Z.); lihua5445@163.com (H.L.); 15762251758@163.com (X.L.)

**Keywords:** *Procambarus clarkii*, oregano essential oil, intestine, gut microbiota

## Abstract

This study used crayfish (*Procambarus clarkii*) as the experimental animal and conducted comparative feeding trials by adding oregano (*Origanum vulgare* L.) essential oil to the diet, systematically revealing its regulatory effects on body weight, muscle quality, intestinal health, and gut microbiota structure. Results showed that oregano essential oil increased the average body weight of crayfish by 12.6%, significantly raised the meat yield from 10.67% to 13.46%, and improved the levels of C22:6n3, cysteine, and umami amino acids in muscle tissue. Additionally, oregano essential oil significantly enhanced intestinal lipase activity, boosted superoxide dismutase and lysozyme activities, optimized gut microbial composition, markedly increased the abundance of beneficial bacteria such as *Bacilli*, and elevated microbial species richness. The study demonstrated that oregano essential oil can simultaneously improve body weight and intestinal lipase activity without disrupting intestinal physiological homeostasis, providing a reference for the application of oregano essential oil in shrimp farming.

## Simple Summary

This study explored whether adding oregano (*Origanum vulgare* L.) essential oil to the feed of crayfish (*Procambarus clarkii*) could improve their body weight, muscle nutritional components, and intestinal health. The results showed that dietary oregano essential oil increased average body weight by 12.6% and meat yield from 10.67% to 13.46%. In muscle tissue of crayfish, we observed elevated levels of C22:6n3, cysteine, and umami-related amino acids. Furthermore, the supplementation was associated with increased intestinal lipase activity, enhanced superoxide dismutase and lysozyme activities, and a shift in gut microbiota composition, characterized by a higher abundance of *Bacilli* and increased microbial species richness. These findings provide empirical data on the physiological responses of crayfish to dietary oregano essential oil, offering a basis for further research into plant-derived feed additives for aquaculture. However, additional studies are needed to assess long-term effects and potential toxicity profiles before practical application.

## 1. Introduction

Oregano essential oil is a natural active compound extracted from oregano (*Origanum vulgare* L.) plants, primarily composed of carvacrol [1] and thymol [2]. It effectively inhibits the growth of various bacteria and fungi, such as *Escherichia coli* [3,4] and *Salmonella* [5], by disrupting the structural integrity of pathogenic microorganisms’ cell membranes. Additionally, oregano essential oil exhibits significant feed-stimulating properties [6]; its aromatic odor stimulates receptors in the animal’s digestive tract, increasing feed intake and digestive enzyme activity, thereby enhancing nutrient absorption and growth performance [7]. Regarding its antioxidant effects, its polyphenolic compounds can scavenge free radicals, protect cells from oxidative damage, and improve overall health [8,9]. These characteristics make it an ideal alternative to antibiotics, particularly favored in green farming and organic agriculture.

Oregano essential oil is rich in phenolic and terpenoid compounds, exhibiting a broad spectrum of biological activities, including anti-inflammatory, antibacterial, antioxidant, and antiviral effects. As a feed additive, it has been shown to improve growth performance and meat quality in aquatic animals [10]. Adding an appropriate amount of oregano essential oil to feed enhances the intestinal barrier function of cyprinid fish, promotes intestinal villus development, and improves digestion and absorption efficiency, thereby boosting growth performance and feed conversion rates [7]. In grass carp farming, oregano essential oil significantly reduces the infection rate of *Aeromonas hydrophila* and alleviates intestinal inflammation [11]. In antibiotic-free farming of *Litopenaeus vannamei*, supplementing feed with oregano essential oil markedly improves growth performance, digestive capacity, immune response, and antioxidant capacity in shrimp, while also increasing the richness and diversity of intestinal microbial flora [12].

The global production of farmed crayfish (*Procambarus clarkii*) reached approximately 3.2 million tons in 2023, ranking second among all cultured crustacean species worldwide. China remains the dominant producer of this crayfish, with its farming area expanding to roughly 5.02 million acres and annual output reaching approximately 3.44 million tons in 2024 [13]. Crayfish has become a widely cultivated species of considerable economic importance. During the farming process of crayfish (*Procambarus clarkii*), diseases frequently occur, seriously threatening both yield and quality. Among these, bacterial enteritis [14], white spot syndrome [15], and black gill disease [16] are particularly prevalent and have become major challenges for farmers. Chinese herbal medicine plays a green and safe role in disease prevention, control, and health promotion in crayfish farming. Its primary function lies in effectively combating common diseases such as bacterial enteritis and white spot syndrome through multiple mechanisms, including antibacterial, antioxidant, and immune-regulating effects, thereby reducing the reliance on antibiotics [17].

Research has shown that dietary supplementation with oregano essential oil optimizes hepatopancreatic function in crayfish through the coordinated regulation of detoxification pathways, protein synthesis, and lipid metabolism networks [18]. Under laboratory conditions, dietary oregano essential oil supplementation at 500 mg/kg for three weeks enhanced antioxidant and immune parameters (enzyme activities and gene expression) and reshaped intestinal microbiota in red swamp crayfish without histologically damaging the hepatopancreas or intestine [19]. Oregano essential oil, an important additive in aquatic animal feed, contains active components that may positively influence crayfish farming. This study evaluates the effects of dietary oregano essential oil on the intestinal health (enzyme activities, tissue morphology, and gut microbiota) and muscle nutritional quality of crayfish in paddy field aquaculture, aiming to clarify its functional role in the farming process.

## 2. Materials and Methods

### 2.1. Experimental Animals and Experimental Design

Ten thousand healthy, uninjured crayfish, each weighing 5.0 ± 0.1 g, were selected. They were randomly distributed into two fish ponds (each approximately 667 m^2^ in area) located in Yibin City, Sichuan Province, with 5000 crayfish assigned to each pond. One pond was fed only the basic feed, whose composition (see Table 1) was based on Wu et al., and this group served as the control. The oregano group received a diet supplemented with oregano essential oil at a dosage of 100 mg/kg, which was determined based on preliminary trials and published data [18]. The essential oil, derived from *Origanum vulgare* L., was supplied by Sichuan Hengrui Tongda Co., Ltd. (Chengdu, China). Product specifications indicated that carvacrol and thymol were the predominant bioactive components. To prepare the experimental diet for the oregano group, the oregano essential oil was first mixed with soybean oil at a ratio of 1:5, and the resulting mixture was then sprayed onto the basal feed to achieve a final concentration of 100 mg oregano phenols per kg of feed. A 60-day feeding trial was then conducted. During the feeding period, environmental parameters, namely temperature at 18–26 °C, dissolved oxygen above 5.0 mg/L, pH between 7.2 and 7.8, and ammonia nitrogen below 0.2 mg/L, were monitored daily and maintained consistently across groups. Feeding took place twice daily (at 8:00 a.m. and 5:00 p.m.), and the daily ration was set at 3% of the crayfish’s body weight.

### 2.2. Sample Collection

After the feeding trial, the crayfish were fasted for 24 h. Twenty-five intact and unharmed crayfish were randomly selected from each pond, weighed individually, and then dissected to collect the tail muscle (the edible part of the tail). The tail muscle was weighed, and the meat yield was calculated using the following formula: meat yield (%) = (tail muscle weight/body weight) × 100%. First, anesthesia was administered using MS-222, followed by dissection. The entire intestinal tract was removed and placed in a cryotube, which was then rapidly frozen in liquid nitrogen and subsequently stored at −80 °C. This procedure was performed for the analysis of intestinal digestive enzymes, immune enzyme activities, microbial composition, metabolome, and transcriptome. Subsequently, the tail muscle was separated, weighed, and stored at −20 °C for the determination of amino acid and fatty acid contents.

### 2.3. Determination of Enzyme Activity Indicators

Five intestinal tissue samples from both the oregano and control groups were stored at −80 °C. Following the instructions and protocols provided by the reagent kit from Quanzhou Ruixin Biotechnology Co., Ltd. (Quanzhou, China), the activities of superoxide dismutase (SOD), malondialdehyde (MDA), catalase (CAT), lysozyme (LZM), alanine aminotransferase (ALT), aspartate aminotransferase (AST), lactate dehydrogenase (LDH), total protease (TP), lipase (LPS), and α-amylase (α-AL) were measured.

### 2.4. Analysis of Amino Acids and Fatty Acids

The contents of 17 hydrolyzed amino acids in muscle tissue of crayfish were determined using high-performance liquid chromatography (HPLC). First, muscle samples were placed in hydrolysis tubes, and 6 mol/L hydrochloric acid was added. The samples were then hydrolyzed at 110 °C for 22–24 h to completely break down proteins into free amino acids. The hydrolysate was dried, re-solubilized, and subjected to a pre-column derivatization reaction using phenyl isothiocyanate (PITC). The derivatized samples were separated using a Thermo Fisher U3000 liquid chromatograph (Thermo Fisher Scientific, Dreieich, Germany), equipped with a reversed-phase C18 chromatographic column, and detected at a wavelength of 254 nm through a gradient elution program. The contents of aspartic acid (Asp), glutamic acid (Glu), serine (Ser), glycine (Gly), histidine (His), arginine (Arg), threonine (Thr), alanine (Ala), proline (Pro), tyrosine (Tyr), valine (Val), methionine (Met), cysteine (Cys), isoleucine (Ile), leucine (Leu), phenylalanine (Phe), and lysine (Lys) were quantified using the external standard method. The fatty acid composition of the muscle was analyzed by gas chromatography (Agilent Technologies Inc., Santa Clara, CA, USA; column: OmegawaxTM 320). Components were identified qualitatively based on the retention times of standard substances and quantified using the peak area normalization method. The contents of saturated fatty acids (SFA), monounsaturated fatty acids (MUFA), and polyunsaturated fatty acids (PUFA) were calculated. This method was based on the description by Li et al. [20].

### 2.5. Intestinal Histology

Intestinal tissues of crayfish were fixed in 4% paraformaldehyde and subsequently dehydrated through a graded ethanol series: 70% ethanol for 4 h, 80% ethanol for 2 h, 90% ethanol for 2 h, 95% ethanol for 2 h, and absolute ethanol for 1 h. This was followed by clearing in xylene for 30 min and infiltration with paraffin at 60 °C for 1.5 h. The tissues were then embedded in paraffin and sectioned at a thickness of 4 μm using a rotary microtome. The sections were dewaxed in xylene and stained with hematoxylin and eosin (H&E) following standard procedures. After dehydration and clearing, the sections were mounted with neutral balsam. Images were captured using a 3DHISTECH Pannoramic SCAN digital slide scanner (3DHISTECH, Budapest, Hungary).

### 2.6. Analysis of Intestinal Microbiota

The intestinal microbiota community structure was analyzed using 16S rRNA gene amplicon sequencing with Illumina 2 × 250 bp paired-end technology. Total DNA from intestinal contents was extracted using a commercial DNA extraction kit, and genomic DNA integrity was assessed by agarose gel electrophoresis. DNA quality was evaluated using a Nanodrop 2000 spectrophotometer (Thermo Fisher Scientific, Wilmington, DE, USA) (concentration ≥ 20 ng/μL, total amount ≥ 500 ng, OD260/280 = 1.8–2.0). For qualified samples, high-fidelity PCR amplification of the target region was performed with five biological replicates, using a standard bacterial/fungal genomic DNA mix as a positive control. The amplification primers were: Primer F = Illumina adapter sequence 1 + CCTACGGGNGGCWGCAG; Primer R = Illumina adapter sequence 2 + GACTACHVGGGTATCTAATCC. After purification and quantification of PCR products, libraries were sequenced on the NovaSeq 6000 platform (Illumina San Diego, CA, USA) using an SP-Xp (PE250) paired-end sequencing strategy, followed by bioinformatics analysis. Data processing was conducted using the DADA2 plugin in QIIME2 (version 2025.10) for quality filtering, denoising, merging, and chimera removal. Taxonomic annotation of amplicon sequence variants (ASVs) was performed with QIIME2 using confidence thresholds of 0.7 for 16S rRNA genes and 0.6 for ITS regions. Species annotation and abundance analyses were based on ASVs. Alpha diversity indices (Chao1, Shannon, Simpson, etc.) and beta diversity distances were calculated, followed by principal coordinate analysis (PCoA) and unweighted pair group method with arithmetic mean (UPGMA) clustering to compare microbial community differences between groups. Analysis of similarities (ANOSIM) was performed using the anosim() function from the vegan package in R (version 2.6-8), with Bray–Curtis dissimilarity selected as the distance metric and the number of permutations set to 9999. Linear Discriminant Analysis Effect Size (LEfSe) was used to screen for taxa that could best explain the inter-group differences. Taxa with significant relative abundance differences were identified using the Kruskal–Wallis rank-sum test, and those with |LDA| > 2 and *p* < 0.05 were retained as discriminants. Results were presented in graphical form. Phylogenetic Investigation of Communities by Reconstruction of Unobserved States (PICRUSt) was employed to predict the metabolic functions of the gut microbiota by referencing 16S rRNA gene sequences against a functional genome database. The software corrects for variations in 16S rRNA gene copy numbers across taxa, enhancing the accuracy of functional predictions from the original abundance data.

### 2.7. Correlation Analysis

To explore the potential links between intestinal enzyme activities and microbial community structure, Spearman’s rank correlation analysis was performed between the relative abundances of dominant bacterial taxa (at the class level). Only correlations with an absolute correlation coefficient (|r|) ≥ 0.3 and a *p*-value < 0.05 were considered statistically significant and retained for visualization. The results were presented as a correlation heatmap.

### 2.8. Data Analysis and Statistics

All indicators underwent preliminary statistical analysis using Excel 2010, with results expressed as mean ± standard deviation (X ± SD). Wilcoxon rank-sum test was used for pairwise comparisons between groups; differences were considered significant at *p* < 0.05. Further statistical analysis was conducted using the standard least squares method in JMP 10.0 software. Significance was assessed using Student’s *t*-test, with differences considered significant at *p* < 0.05.

## 3. Results

### 3.1. Body Weight and Tail Meat Yield

As shown in Table 1, after adding oregano essential oil to the feed, the average weight of the treated crayfish group reached 26.59 ± 4.02 g, which was significantly higher than the control group’s average weight of 23.61 g (*p* < 0.05), representing a 12.6% increase. The average tail muscle weight of the treated group was 3.55 g, with a meat yield of 13.46%, both significantly higher than the control group’s average tail muscle weight of 2.47 g and meat yield of 10.67% (*p* < 0.05).

### 3.2. The Content of Amino Acids and Fatty Acids in Muscles

**Figure 1:**
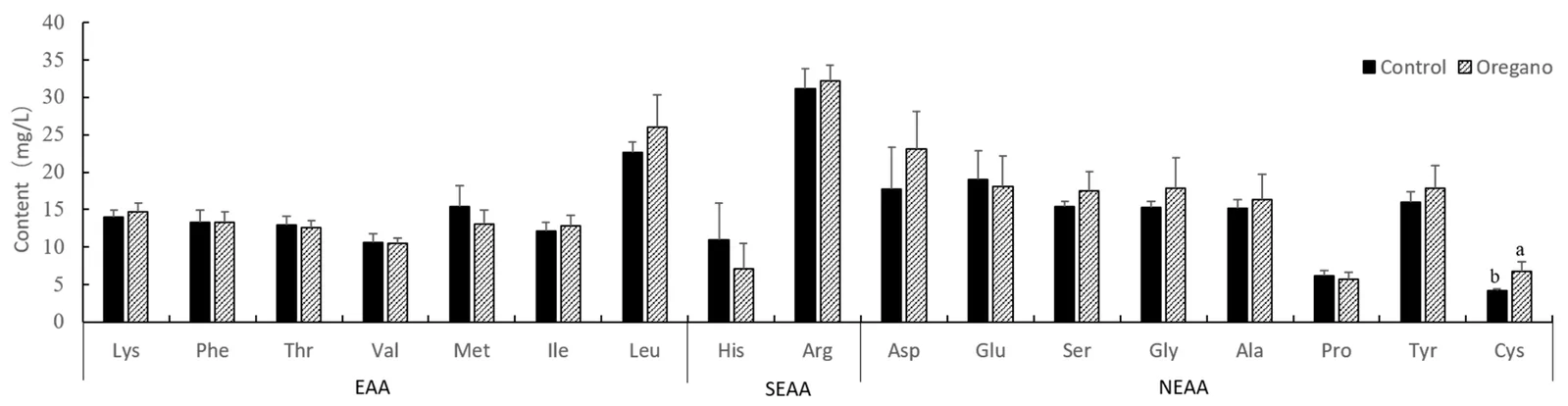
Amino acid content in the muscle of crayfish. Different lowercase letters indicate significant differences between groups (*p* < 0.05, *n* = 5).

The results showed that 17 types of amino acids were detected in the muscles of both groups of crayfish (Figure 1, Table S1). These included 7 essential amino acids (EAAs), 2 semi-essential amino acids (SEAAs), and 8 non-essential amino acids (NEAAs). The cysteine content in the muscle of the oregano group was significantly higher than that of the control group (*p* < 0.05), while the levels of the other 16 amino acids did not differ significantly between the oregano and control groups (*p* > 0.05). Among these amino acids, the concentrations of the umami amino acids aspartic acid, glycine, and alanine were slightly higher in the oregano group compared to the control group.

By analyzing 37 types of fatty acids, 25 were detected in the tail muscle of crayfish in both the oregano and control groups (Table 2). These included 13 saturated fatty acids, 4 monounsaturated fatty acids, and 8 polyunsaturated fatty acids. Notably, the levels of unsaturated fatty acids C18:1n9c, C18:2n6c, C18:3n3, and C20:5n3 were significantly higher than those of saturated fatty acids. The concentrations of saturated fatty acids C14:0, C15:0, C17:0; monounsaturated fatty acid C16:1; and polyunsaturated fatty acids C18:3n6, C18:3n3, and C20:3n3 in the oregano group’s muscle were significantly lower than those in the control group (*p* < 0.05). No significant differences were observed in other fatty acids between the two groups (*p* > 0.05). Additionally, the content of C22:6n3 (DHA) per 100 g of muscle was higher in the oregano group (2.12 mg) compared to the control group (1.45 mg).

### 3.3. Intestinal Enzyme Activity and Tissue Structure

The LPS content in the intestinal tissues of the treated crayfish group was 16.47 nmol/mg, which was significantly higher than that of the control group (10.49 nmol/mg) (*p* < 0.05). The TP and α-AL activities in the intestinal tissues of the treated group were also higher than those in the control group; however, these differences were not statistically significant (*p* > 0.05) (Table 3).

The contents of SOD and LZM in the intestinal tissues of the crayfish oregano group were 2.89 U/mg and 10.09 nmol/mg, respectively, which were significantly higher than those in the control group (1.68 U/mg and 8.66 nmol/mg) (*p* < 0.05). The addition of oregano essential oil to the feed had no significant effect on the contents of MDA, CAT, AST, ALT, and LDH in the intestinal tissues (*p* > 0.05) (Table 4).

**Figure 2:**
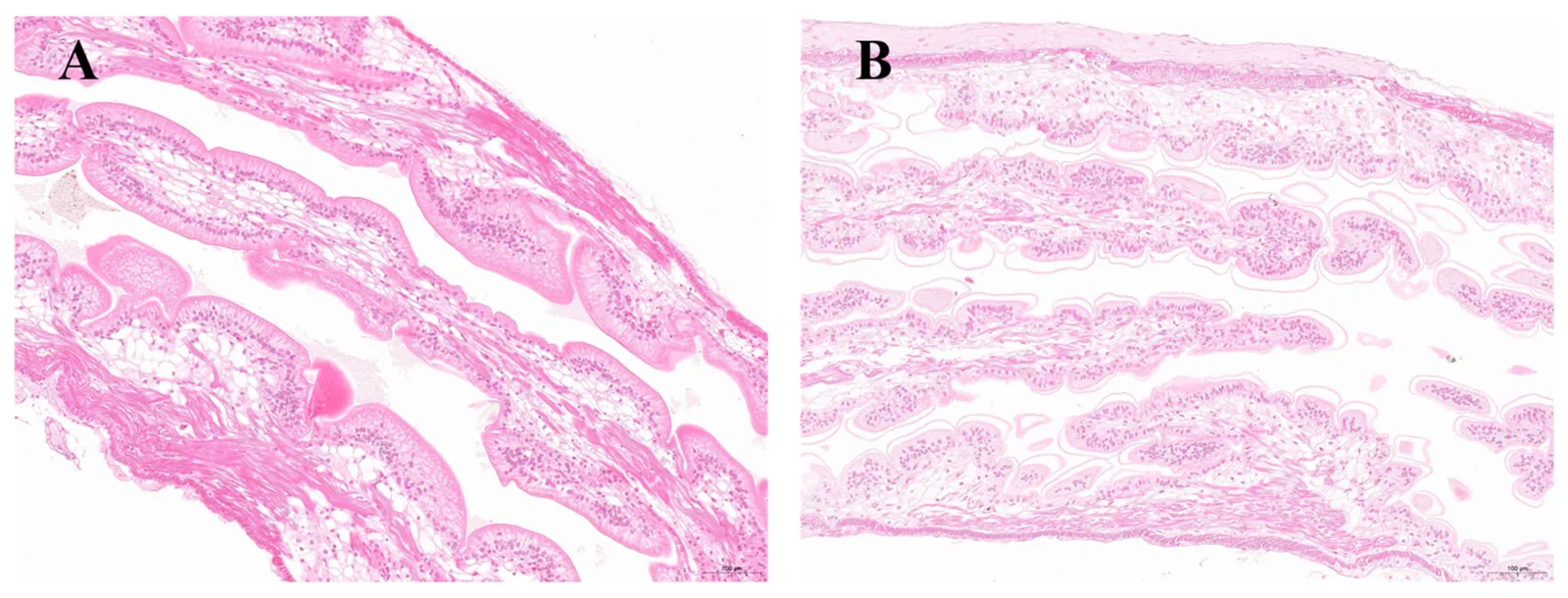
H&E stain of intestinal tissues from the control group (**A**) and oregano group (**B**) of crayfish (100 μm, 20×).

The mucosal epithelial cells of the intestine in control and oregano groups were arranged in an orderly manner, and the submucosa and muscularis layers exhibited essentially normal histological architecture, with no obvious inflammatory cell infiltration observed. In the oregano group, the intestinal villi were slender and evenly spaced, projecting freely into the lumen, whereas those in the control group appeared relatively short and thick, with a broadened morphology and numerous intracellular vacuoles (Figure 2).

### 3.4. The Composition of Intestinal Microorganisms

**Figure 3:**
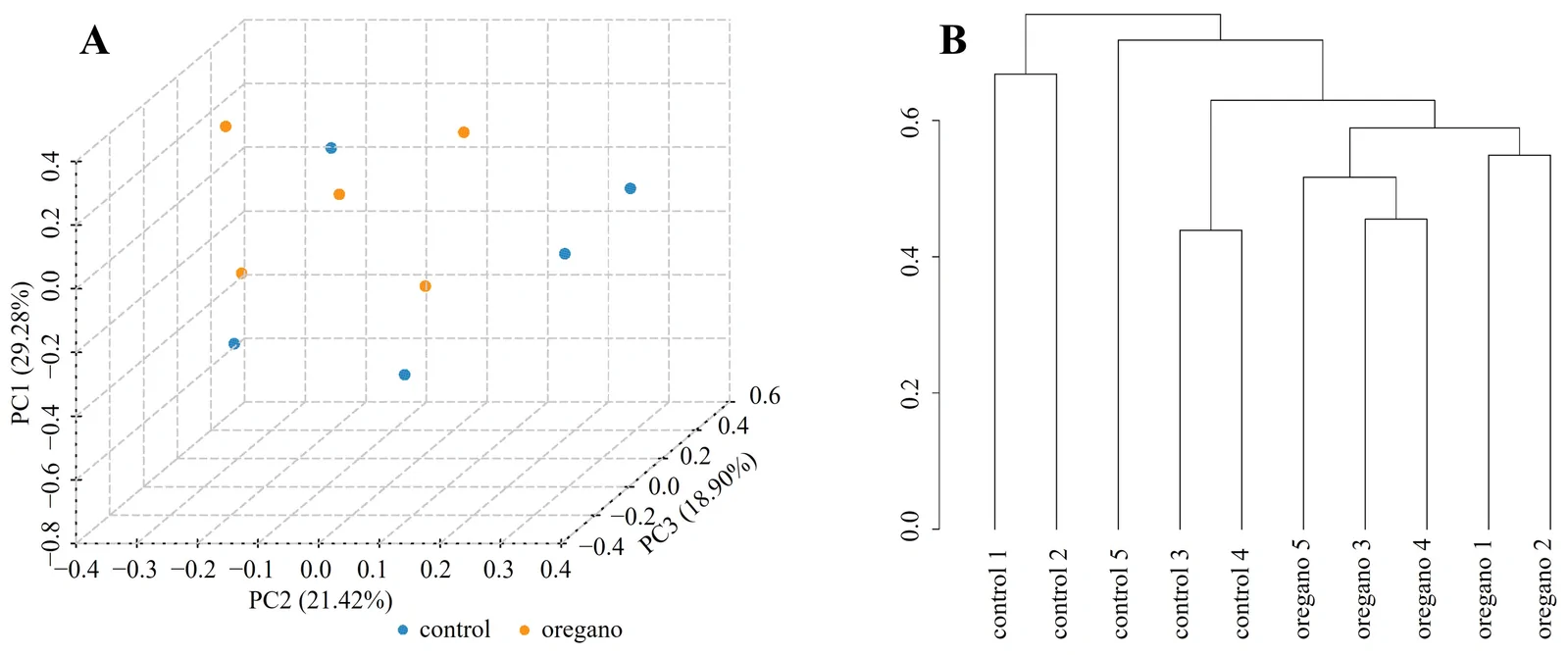
PCoA (**A**) and UPGMA cluster analysis (**B**). The length of the branches represents the distance between the samples, and the closer the branches are to each other, the more similar the species composition of the samples (*n* = 5).

Through relative quantitative sequencing of microbial 16S amplicons, 1834 ASVs were detected in the intestinal tracts of the oregano-treated crayfish group, while 1322 ASVs were identified in the control group, with 284 ASVs shared between both groups. Beta diversity was assessed using PCoA and further evaluated by UPGMA hierarchical clustering. The PCoA score plot revealed that samples from the control and oregano groups occupied distinct regions of the ordination space. ANOSIM was performed to assess the validity of the grouping by testing whether inter-group differences were significantly larger than intra-group differences. The analysis yielded an R-statistic of 0.368 and a *p*-value of 0.024. The positive R-value indicated that within-group distances were smaller than between-group distances, confirming the effectiveness of the current grouping scheme, and the *p*-value below 0.05 demonstrated statistical significance. Consistently, the UPGMA dendrogram showed that samples clustered primarily by oregano rather than by random variation, further corroborating the compositional divergence identified by PcoA (Figure 3A,B).

**Figure 4:**
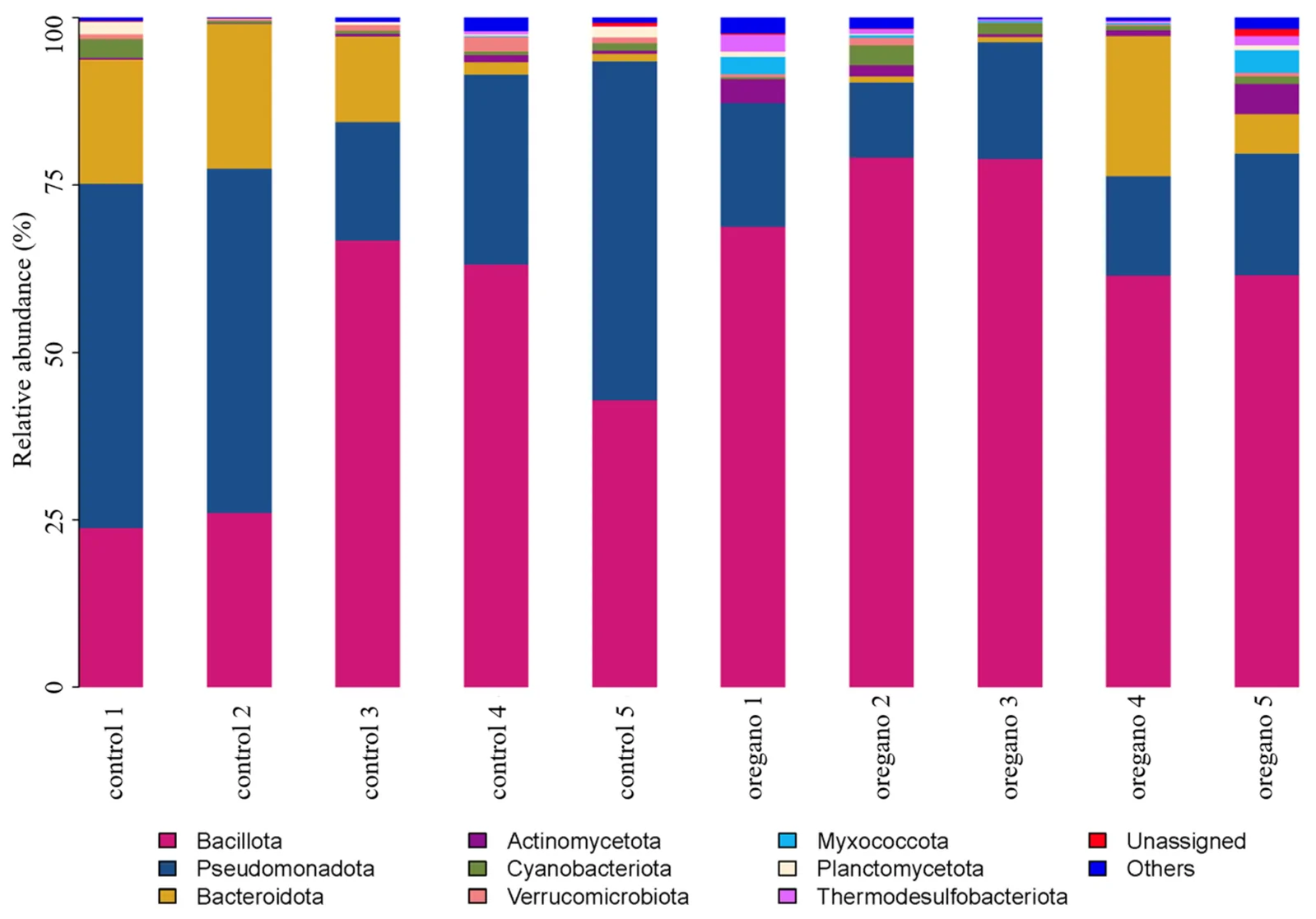
Relative abundance of intestinal microbiota at the phylum level (*n* = 5).

When analyzed at the genus level, the dominant bacterial communities in the intestines of crayfish from both the oregano and control groups were relatively similar, with the main groups being Bacillota, Pseudomonadota, and Bacteroidota (Figure 4). In terms of relative abundance, the average proportion of B Bacteroidota in the oregano group was 70.00%, which was higher than the 44.53% observed in the control group; however, this difference was not statistically significant (*p* > 0.05). The average relative abundances of Pseudomonadota and Bacteroidota in the oregano group were 16.01% and 5.70%, respectively, both lower than the 39.90% and 11.20% observed in the control group.

**Figure 5:**
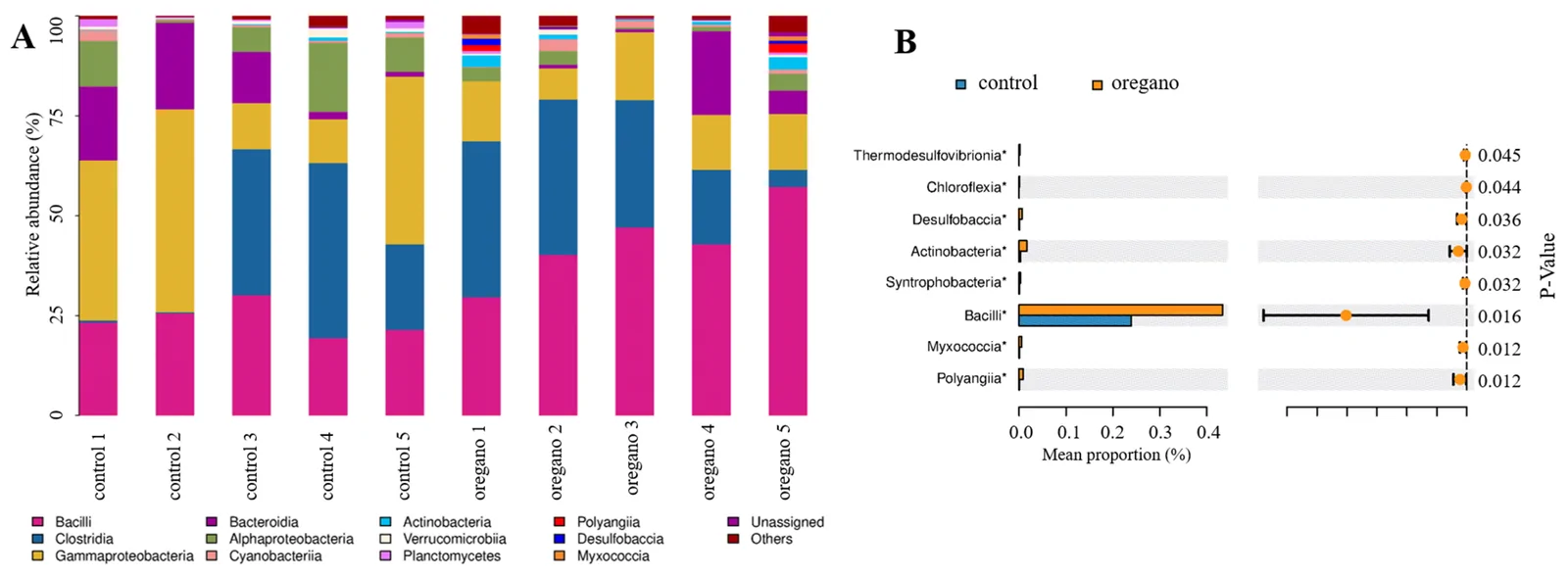
Relative abundance of gut microbes at class level (**A**) and differentially abundant genera between two groups at class level by Wilcoxon rank-sum test analysis (**B**). Only data with significant differences (*p* < 0.05) between groups are shown (*n* = 5). * indicates a significant difference between groups (*p* < 0.05).

At the class level, the bacterial community composition in the intestinal tracts of crayfish was relatively similar between the oregano and control groups. Both groups were predominantly composed of Bacilli, Clostridia, Gammaproteobacteria, and Bacteroidia (Figure 5A). The average relative abundance of Bacilli in the oregano group was 43.39%, which was significantly higher than the 23.96% observed in the control group (*p* < 0.05) (Figure 5B). The oregano group also exhibited a higher average relative abundance of Clostridia (26.57%) compared to the control group (20.53%). In contrast, the average relative abundances of Gammaproteobacteria and Bacteroidia in the oregano group were 13.48% and 5.70%, respectively, both lower than the 31.07% and 11.20% observed in the control group.

**Figure 6:**
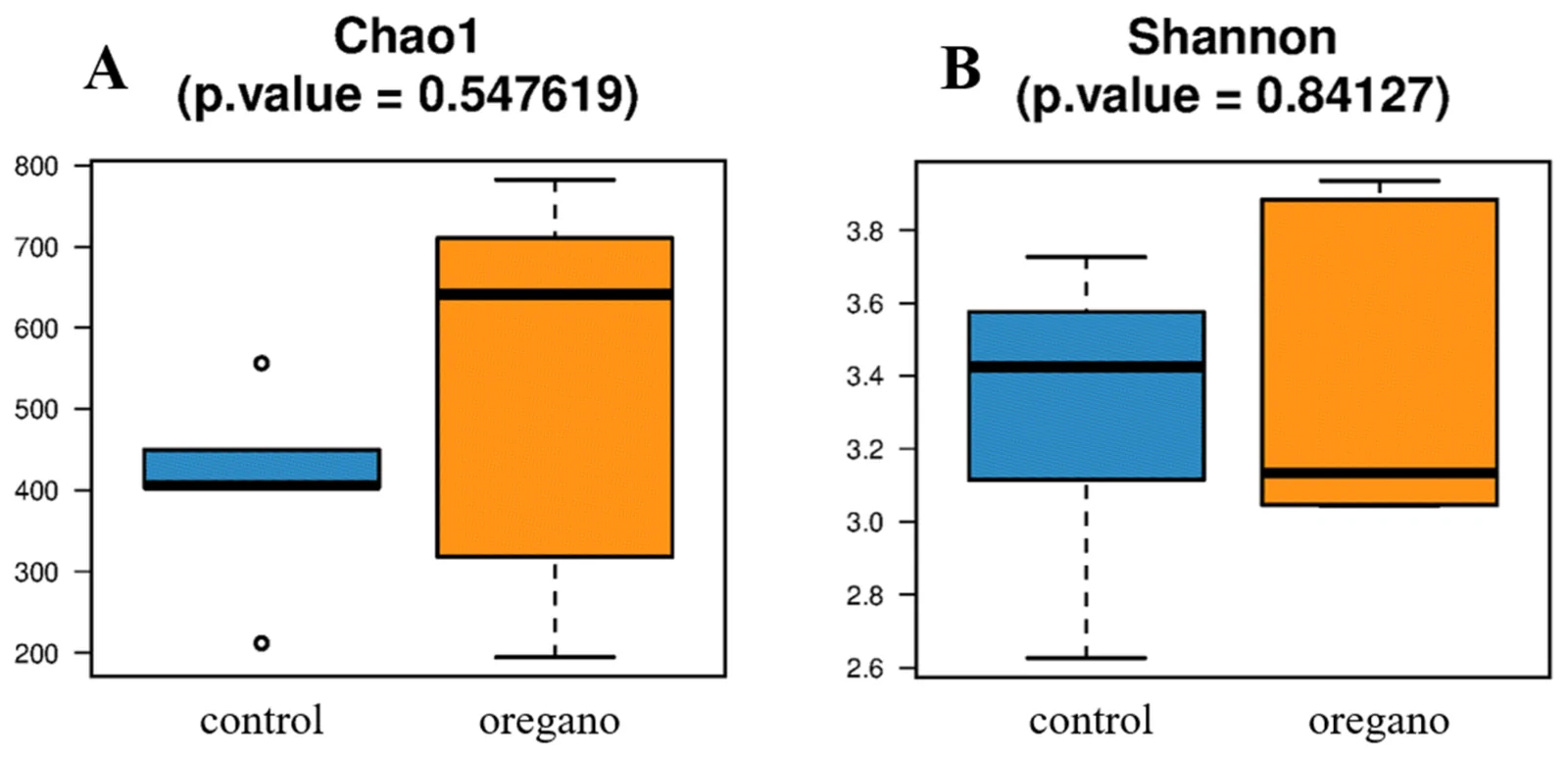
Analysis of α diversity of intestinal microbiota using Chao1 (**A**) and Shannon index (**B**).

The alpha diversity analysis of the intestinal microbiota in crayfish showed that the Chao1 index was 405.36 in the control group and 529.32 in the oregano group (Figure 6A). The Shannon index was 3.29 for the control group and 3.41 for the oregano group. There were no significant differences in either the Chao1 or Shannon indices between the two groups (*p* > 0.05) (Figure 6B).

**Figure 7:**
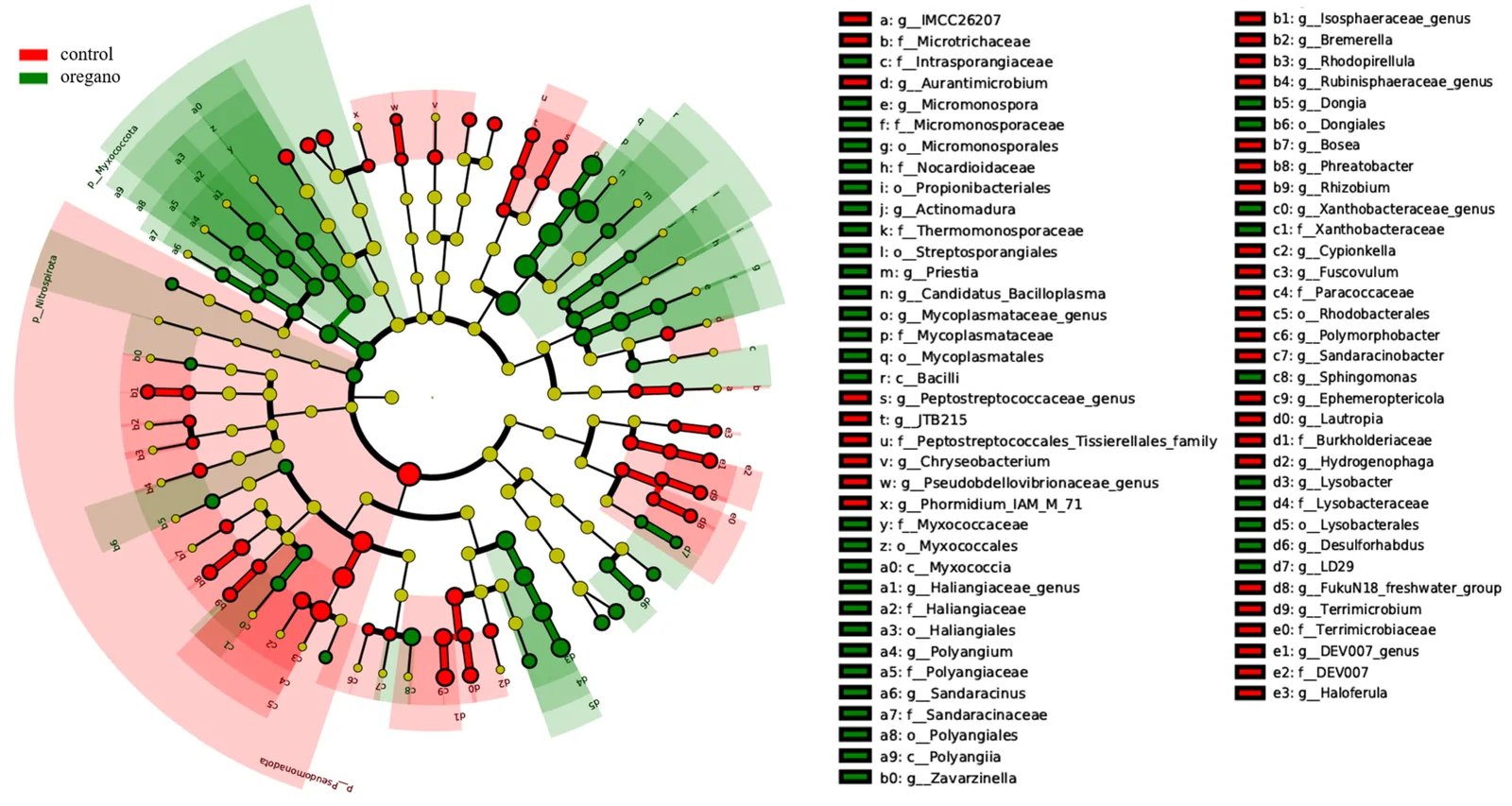
Impact of oregano essential oil on the intestinal flora: LefSe analysis (*n* = 5). LEfSe cladogram showing taxonomic differences in the intestinal microbiota between the control and oregano groups. Each node represents a taxon at a given phylogenetic level, with letters (a–e3) indicating taxa that exhibited significant differences (LDA > 2, *p* < 0.05).

LEfSe analysis was performed to identify bacterial taxa with significantly different relative abundances between the control and oregano groups (Figure 7). At the phylum level, the cladogram revealed that the oregano group was characterized by a higher abundance of taxa belonging to Actinobacteria (including Micromonosporaceae, Nocardioideaceae, and Thermomonosporaceae) and Firmicutes (including Bacilli, Mycoplasmataceae, and Peptostreptococcaceae). In contrast, the control group showed enrichment of taxa affiliated with Bacteroidetes (Chryseobacterium, Sandaracinaceae) and Planctomycetes (Rhodopirellula, Dongia, Haloferula).

**Figure 8:**
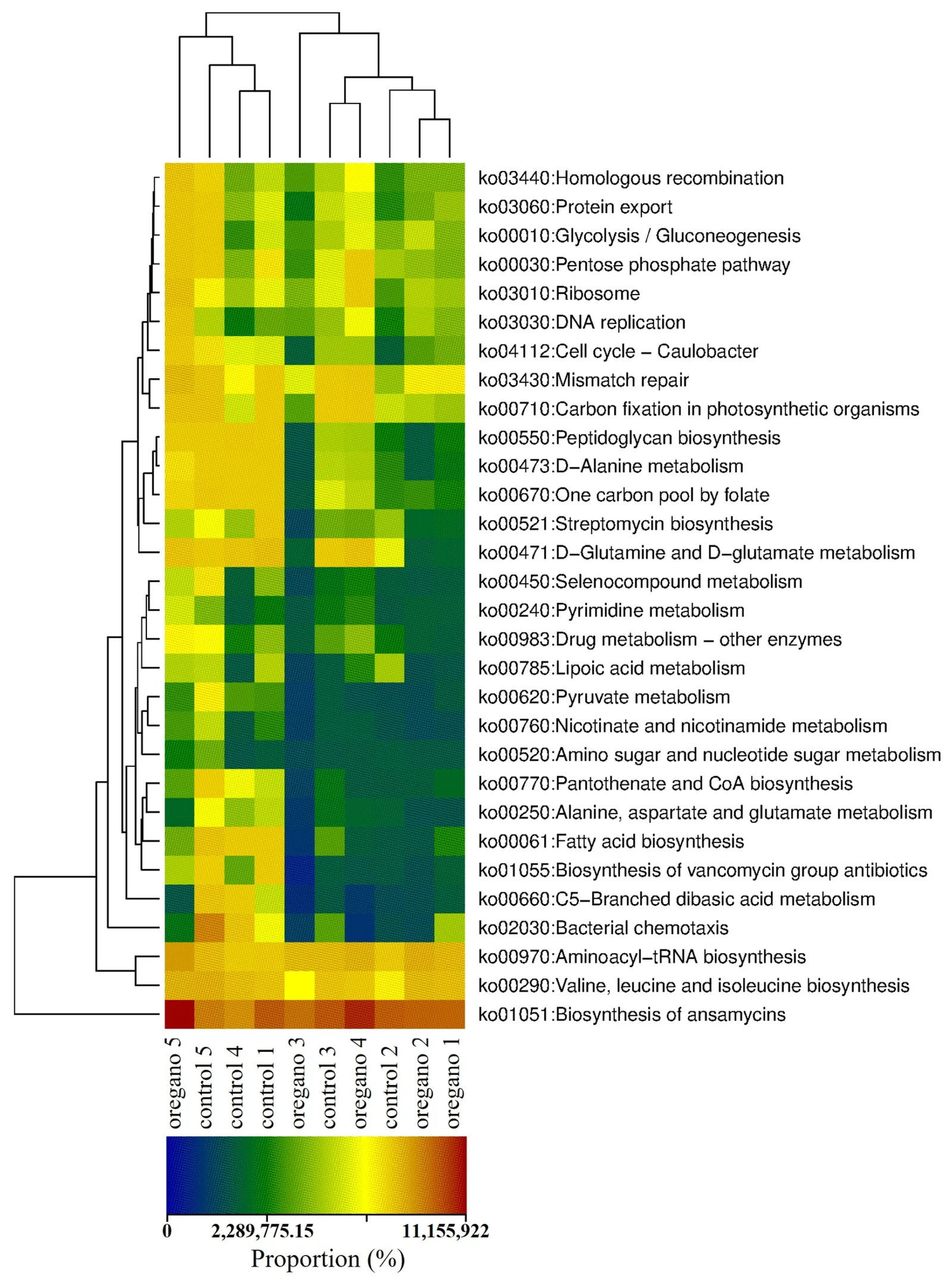
Heatmap of KEGG pathway abundances (*n* = 5). The color gradient from light to dark indicates increasing relative abundance of the corresponding functions.

PICRUSt2 was employed to predict the functional profile of the gut microbiota (Figure 8). Compared with the control group, the oregano group exhibited higher relative abundances of pathways involved in carbohydrate metabolism, including glycolysis/gluconeogenesis (ko00010), the pentose phosphate pathway (ko00030), pyruvate metabolism (ko00620), and amino sugar and nucleotide sugar metabolism (ko00520). Several amino acid metabolism pathways, such as alanine, aspartate, and glutamate metabolism (ko00250), valine, leucine, and isoleucine biosynthesis (ko00290), and D-glutamine and D-glutamate metabolism (ko00471), showed enrichment in the oregano group. In contrast, the oregano group displayed reduced abundances of pathways associated with peptidoglycan biosynthesis (ko00550), bacterial chemotaxis (ko02030), and the biosynthesis of streptomycin (ko00521) and vancomycin group antibiotics (ko01055).

**Figure 9:**
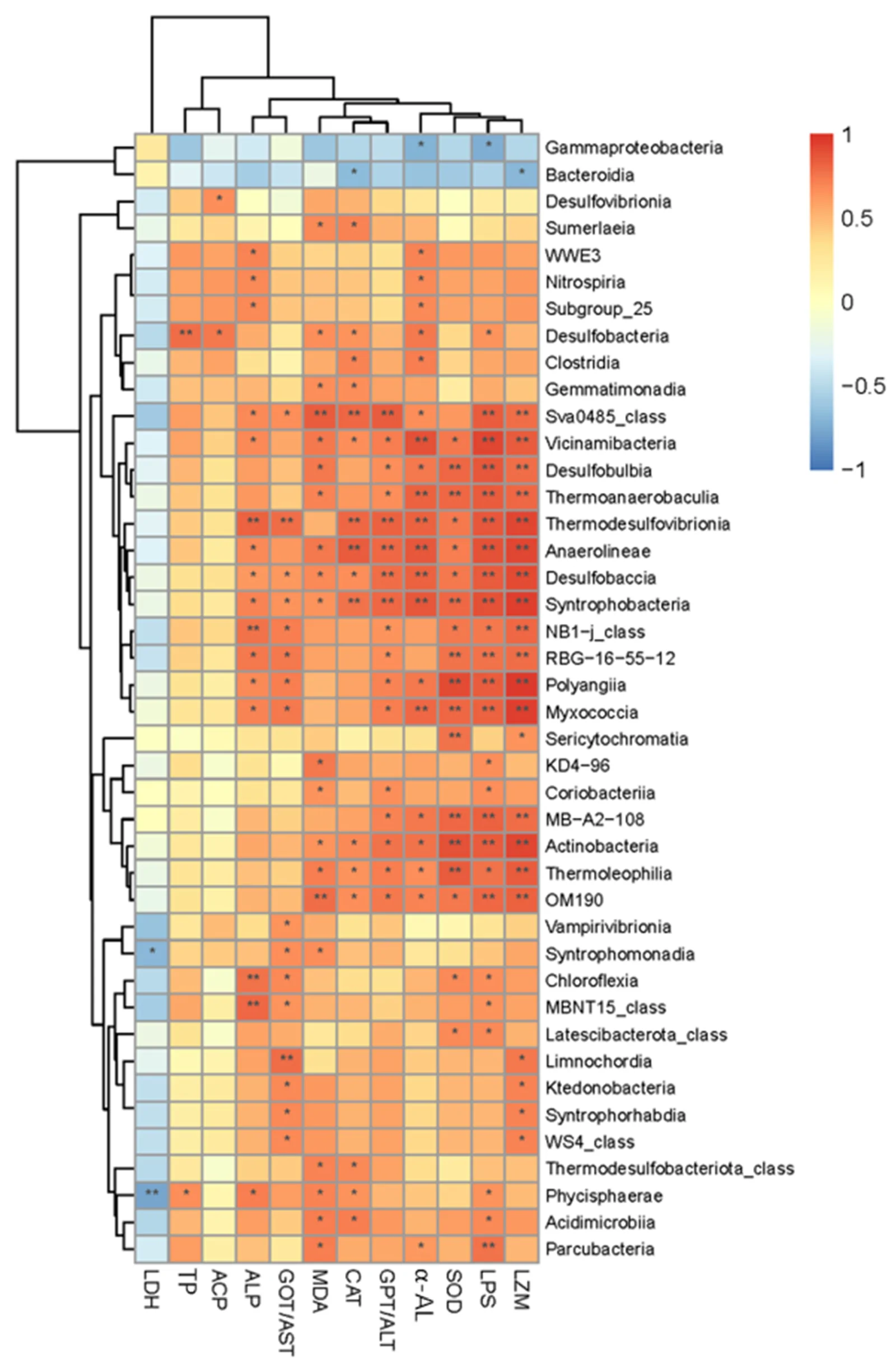
Correlation analysis of gut microbiota (at the class level) and intestinal enzyme activities (*n* = 5). The red and blue colors indicate positive and negative correlations, respectively. * indicates significant differences (*p* < 0.05), ** indicates significant differences (*p* < 0.01).

The correlation heatmap revealed multiple significant associations between intestinal enzyme activities and specific microbial taxa at the class level. Regarding digestive enzymes, LPS exhibited positive correlations with the relative abundances of Desulfobacteria, Vicinamibacteria, and Desulfobulbia, etc., while a negative correlation was observed with Gammaproteobacteria. For immune-related enzymes, LZM was positively associated with Vicinamibacteria, Desulfobulbia, and Thermoanaerobaculia, etc., whereas a negative correlation was found with Bacteroidia. Regarding antioxidant indices, SOD showed positive correlations with Vicinamibacteria, Desulfobulbia, and Thermoanaerobaculia, etc. (Figure 9).

## 4. Discussion

### 4.1. Effects of Oregano Essential Oil on Growth Performance and Meat Yield

Herbal extracts, when used as feed additives, have been shown to promote growth, enhance intestinal digestive function, and improve immune capacity in aquatic animals [21,22]. Oregano essential oil, as a natural immunostimulant of plant origin, exhibits multiple biological activities, including antibacterial, anti-inflammatory, anti-ulcer, anti-stress, and growth-promoting effects [23]. Numerous studies have demonstrated the positive effects of oregano essential oil on the growth performance of aquatic organisms. For instance, dietary supplementation with oregano essential oil significantly improved growth performance and intestinal morphometry in carp fingerlings [7]. Rashidian et al. [24] confirmed the growth-promoting effects of oregano extract in zebrafish. Stylianaki et al. [25] and Dang et al. [26] further reported that dietary oregano essential oil supplementation also significantly enhanced growth performance in rainbow trout. In shrimp, dietary inclusion of 0.6% oregano polyphenols significantly improved growth performance and non-specific immunity in Pacific white shrimp (*Litopenaeus vannamei*) [27]. In this study, dietary oregano essential oil supplementation significantly increased body weight, tail muscle weight, and meat yield of crayfish (*p* < 0.05). The results suggest that oregano essential oil may exert a positive growth-promoting effect on crayfish, which is consistent with the results observed in the aforementioned aquatic species.

### 4.2. Effects of Oregano Essential Oil on Muscle Amino Acid and Fatty Acid Composition

The amino acid analysis revealed no significant differences in the content of 16 amino acids between control and oregano groups (*p* > 0.05), without the Cys. However, the levels of the taste-active amino acids (aspartic acid, glycine, and alanine) were slightly higher in the oregano group than in the control group. This result indicates that dietary Oregano essential oil supplementation, while significantly promoting growth, did not fundamentally alter the basic amino acid composition of muscle, which is of great significance for maintaining the nutritional quality of crayfish products. In addition, studies have found that adding Oregano essential oil to crayfish feed can significantly increase the glutathione (GSH) content in the hepatopancreas of crayfish [19]. GSH is a core molecule of the cellular antioxidant defense system, and Cys is an important precursor amino acid for GSH synthesis [28]. In this study, the significant increase in cysteine content in the muscle of crayfish in the Oregano essential oil-supplemented group (*p* < 0.05) is speculated to provide more substrate for GSH biosynthesis, thereby enhancing the host’s antioxidant defense system through the regulation of sulfur-containing amino acid metabolism.

Analysis of the fatty acid content in crayfish muscle revealed that the contents of various saturated fatty acids (C14:0, C15:0, C17:0), monounsaturated fatty acids (C16:1), and polyunsaturated fatty acids (C18:3n6, C18:3n3, C20:3n3) in the oregano group were significantly lower than those in the control group (*p* < 0.05). The major active components of oregano essential oil, carvacrol and thymol, have been reported to regulate the expression of lipid metabolism-related genes and promote fatty acid β-oxidation and energy utilization [29]. Therefore, it is speculated that under the growth-promoting effect of oregano essential oil, some fatty acids may be preferentially oxidized and utilized as energy substrates, which may be one reason for their decreased content. Increasing the proportion of long-chain polyunsaturated fatty acids is crucial for cell membrane fluidity and immune function in crustaceans [30]. In this study, the contents of C18:1n9c and DHA in the oregano group were slightly higher than those in the control group, suggesting that oregano essential oil may have certain effects on lipid metabolism in crayfish. Studies by Stylianaki et al. [25] in rainbow trout also found that oregano essential oil supplementation could affect the fatty acid profile of fillets, further supporting the regulatory role of oregano essential oil on lipid metabolism. However, the effects of oregano essential oil on muscle nutrients may be species-specific and dose-dependent. Future studies should combine analysis of lipid metabolism-related gene expression and fatty acid synthase activity assays to elucidate the precise molecular mechanisms by which oregano essential oil regulates fatty acid metabolism in crayfish.

### 4.3. Effects of Oregano Essential Oil on Intestinal Digestive Enzyme Activities and Tissue Morphology

Oregano essential oil has been shown to promote nutrient digestion and absorption in aquatic animals [23], and dietary supplementation with oregano essential oil can effectively improve digestive enzyme activities and intestinal morphology in aquatic species [31]. The results of this study showed that oregano essential oil supplementation significantly increased intestinal LPS activity in crayfish (*p* < 0.05), while TP and α-AL activities showed increasing trends but did not reach statistical significance (*p* > 0.05). This result is partially consistent with the observation by Guo et al. [19] that total protease activity in the hepatopancreas of crayfish increased with increasing doses of oregano essential oil. However, no significant change in protease activity was detected in intestinal tissue in this study, which is speculated to be due to tissue specificity. The hepatopancreas is the main site of digestive enzyme synthesis in crustaceans and may be more sensitive to nutritional regulation, whereas the intestine is more focused on the final completion of digestion and absorption. The significant increase in lipase activity has important nutritional implications, as lipids are crucial energy sources and carriers of essential fatty acids for crustaceans [32]. The fatty acid analysis data in this study showed decreased contents of various fatty acids in the muscle of the treatment group, while increased intestinal lipase activity may have promoted the digestive absorption efficiency of dietary fats, thereby improving growth performance.

The intestinal histological results revealed that after oregano essential oil supplementation, the intestinal villi of crayfish became slender and uniformly arranged, protruding freely into the intestinal lumen, whereas the villi in the control group were relatively short and thick, with a broader morphology and more intracellular vacuoles. The improved intestinal villus morphology in crayfish helps increase the contact area between nutrients and the intestinal epithelium, thereby enhancing absorption efficiency [33]. However, Guo et al. [19] did not observe significant changes in villus height in a 3-week laboratory tank feeding trial with oregano essential oil supplementation in crayfish feed, suggesting that differences in rearing period and environmental conditions may have contributed to the discrepancies in experimental results.

### 4.4. Effects of Oregano Essential Oil on Intestinal Immune and Antioxidant Enzyme Activities

This study found that oregano essential oil supplementation significantly increased the activities of SOD and LZM in the intestine (*p* < 0.05), while changes in MDA content, CAT activity, and liver function-related enzymes (AST, ALT, LDH) did not reach significant levels (*p* > 0.05). The significant increases in SOD and LZM reflect, from different aspects, the effects of oregano essential oil on the antioxidant capacity and non-specific immunity of crayfish intestine. Guo et al. [19] found that dietary oregano essential oil significantly increased SOD activity in the hemolymph of crayfish, which is consistent with the significant increase in intestinal SOD activity observed in this study, suggesting that oregano essential oil may have the ability to enhance antioxidant enzyme activities in crayfish. This enhancement of antioxidant enzyme activity may be attributed to the activation of the *Nrf2/Keap1* signaling pathway by phenolic compounds in oregano essential oil, thereby upregulating the expression of antioxidant enzyme genes [34,35]. In addition, Guo et al. [19] detected a significant increase in total antioxidant capacity (T-AOC) in the hepatopancreas of crayfish fed oregano essential oil, whereas no significant changes in CAT and MDA activities were observed in intestinal tissue in this study. It is speculated that this difference may be due to tissue-specific responses to oregano essential oil. The hepatopancreas, as the main metabolic and detoxification organ in crustaceans, may have a stronger inducible antioxidant defense system, while the intestine, as the first digestive organ in direct contact with feed additives, may exhibit different kinetic characteristics in its antioxidant response. In the study by Guo et al. [19], the most significant antioxidant effect was observed at a dose of 500 mg/kg oregano essential oil, while the 100 mg/kg dose used in this study may be within a suboptimal induction range for intestinal antioxidant enzyme activities. This may also explain why only SOD reached significance in this study, while CAT showed an increasing trend but did not reach a significant level.

### 4.5. Effects of Oregano Essential Oil on Intestinal Microbial Communities

Studies have shown that oregano essential oil can regulate the intestinal microbial communities of aquatic animals [26,36]. In this study, 16S rRNA gene sequencing analysis revealed the regulatory effects of oregano essential oil supplementation on the intestinal microbiota of crayfish. Beta diversity analysis showed a significant separation in community structure between the treatment and control groups (R = 0.368, *p* = 0.024), indicating that oregano essential oil, as an exogenous feed additive, may reshape the intestinal microbiota. In terms of alpha diversity, although both Chao1 and Shannon indices showed increasing trends in the treatment group, they did not reach statistical significance (*p* > 0.05). This is not entirely consistent with the findings of Guo et al. [19], who observed that oregano essential oil significantly increased alpha diversity indices in crayfish. It is speculated that this discrepancy may be due to the more complex initial microbiota background and higher environmental microbial exposure in the paddy field culture environment used in this study, which may have masked the enhancing effects of oregano essential oil on diversity. In addition, the absence of replicated paddy field units in this study may have led to biased results, and future studies need to further consider the influence of environmental factors on intestinal microorganisms. Moreover, the dose of oregano essential oil in this study (100 mg/kg) was lower than the doses (300 and 500 mg/kg) that significantly increased diversity indices in the study by Guo et al. [19], and the dosage difference may also be an important factor affecting intestinal microbial communities.

In terms of community composition, this study found that the relative abundance of the class Bacilli in the intestine of the oregano group was significantly higher than that in the control group (43.39% vs. 23.96%, *p* < 0.05). Bacilli contains a large number of genera with probiotic potential, many of which can produce antimicrobial substances, secrete digestive enzymes, and modulate host immunity [37]. Studies have shown that oregano essential oil has strong inhibitory effects on Gram-negative bacteria, while its selective inhibition against some Gram-positive bacteria and Actinobacteria is weaker. In this study, LefSe analysis revealed that the oregano group was enriched in multiple families within the phylum Actinobacteria (Micromonosporaceae, Nocardioideaceae, Thermomonosporaceae) and the class Bacilli, Mycoplasmataceae, and Peptostreptococcaceae within the phylum Firmicutes; the control group was enriched in the genus Chryseobacterium of the phylum Bacteroidetes, Sandaracinaceae, and Rhodopirellula, Dongia, and Haloferula of the phylum Planctomycetes.

PICRUSt2 functional prediction showed that the intestinal microbiome in the oregano group had increased relative abundances of carbohydrate metabolism pathways (glycolysis/gluconeogenesis, pentose phosphate pathway, pyruvate metabolism, amino sugar and nucleotide sugar metabolism) and various amino acid metabolism pathways (alanine/aspartate/glutamate metabolism, valine/leucine/isoleucine biosynthesis, etc.). This functional prediction is consistent with the observed improvements in growth performance and digestive enzyme activities in this study, suggesting that the optimized metabolic functions of the intestinal microbiota may synergize with the host digestive system to enhance nutrient utilization efficiency. Studies by Zhang et al. [7] in koi carp also found that oregano essential oil could modulate intestinal microbiota structure and enhance digestive enzyme activities, further supporting the hypothesis that oregano essential oil improves nutrient metabolism through gut microbiota–host interactions.

This study further explored the potential correlations between intestinal microbiota and enzyme activities through correlation analysis. The results showed that LPS activity was positively correlated with the relative abundances of multiple taxa such as Desulfobacteria, Vicinamibacteria, and Desulfobulbia, but negatively correlated with Gammaproteobacteria; LZM was positively correlated with Vicinamibacteria, Desulfobulbia, and other taxa, and negatively correlated with Bacteroidia; SOD also showed positive correlations with Vicinamibacteria, Desulfobulbia, and other taxa. Although these correlations do not prove causality, they suggest that specific microbial taxa may play important roles in regulating host digestion, immunity, and antioxidant functions. This is logically consistent with the findings of Guo et al. [19], who reported that genera such as PeM15 and Hyphomicrobium were positively correlated with antioxidant and immune gene expression, suggesting that oregano essential oil may indirectly regulate host physiological functions by reshaping the intestinal microbiota structure.

## 5. Conclusions

In conclusion, dietary supplementation with 100 mg/kg oregano essential oil can significantly increase body weight and tail meat yield in crayfish, improve intestinal tissue morphology, enhance digestive enzyme (LPS) activity and immune-antioxidant enzyme (SOD, LZM) activities, and reshape the intestinal microbial community structure. This study provides experimental evidence for the application of oregano essential oil in crayfish aquaculture and lays a foundation for further investigation into its mechanisms of action.

## Figures and Tables

**Table 1:** The weight and tail meat yield of crayfish (*n* = 25).

Groups	Body Weight (g)	Muscle Weight (g)	Meat Yield (%)
Control	23.61 ± 5.22 ^b^	2.47 ± 0.66 ^b^	10.67 ± 2.44 ^b^
Oregano	26.59 ± 4.02 ^a^	3.55 ± 0.66 ^a^	13.46 ± 2.15 ^a^

Note: Different lowercase letters indicate significant differences between groups (*p* < 0.05).

**Table 2:** The fatty acid content in crayfish muscle (*n* = 5).

Type	Index	Control Group (mg/100 g)	Oregano Group (mg/100 g)
SFA	C10:0	0.089 ± 0.033	0.133 ± 0.133
	C11:0	0.027 ± 0.01	0.025 ± 0.005
	C12:0	0.167 ± 0.055	0.129 ± 0.025
	C13:0	0.095 ± 0.003	0.091 ± 0.004
	C14:0	0.361 ± 0.081 ^a^	0.245 ± 0.072 ^b^
	C15:0	0.445 ± 0.061 ^a^	0.311 ± 0.043 ^b^
	C16:0	4.575 ± 0.886	3.587 ± 0.917
	C17:0	0.599 ± 0.031 ^a^	0.389 ± 0.071 ^b^
	C18:0	4.276 ± 0.457	3.747 ± 0.763
	C20:0	0.572 ± 0.07	0.495 ± 0.064
	C21:0	0.295 ± 0.028	0.26 ± 0.021
	C22:0	0.535 ± 0.093	0.457 ± 0.096
	C23:0	0.267 ± 0.009	0.254 ± 0.014
MUFA	C16:1	1.664 ± 0.281 ^a^	0.761 ± 0.483 ^b^
	C20:1	0.307 ± 0.036	0.321 ± 0.037
	C18:1n9c	7.579 ± 1.81	9.293 ± 3.146
	C22:1n9	0.723 ± 0.122	0.6 ± 0.187
PUFA	C18:2n6c	7.285 ± 2.651	6.4 ± 2.205
	C18:3n6	0.293 ± 0.03 ^a^	0.231 ± 0.017 ^b^
	C18:3n3	12.382 ± 5.701 ^a^	1.383 ± 0.467 ^b^
	C20:2	0.449 ± 0.045	0.46 ± 0.092
	C20:4n6	3.448 ± 0.616	2.666 ± 1.819
	C20:3n3	0.668 ± 0.186 ^a^	0.28 ± 0.031 ^b^
	C20:5n3	8.829 ± 2.005	7.413 ± 5.496
	C22:6n3	1.452 ± 0.427	2.12 ± 0.789

Note: Data are presented as mean ± SD. Different lowercase letters within the same row indicate significant differences between groups (*p* < 0.05).

**Table 3:** Content of digestive enzymes in the intestines of crayfish (*n* = 5).

Groups	TP (U/mg)	LPS (nmol/mg)	α-AL (μg/mg)
Control	0.58 ± 0.19	10.49 ± 3.35 ^b^	12.39 ± 3.64
Oregano	0.64 ± 0.16	16.47 ± 4.58 ^a^	17.26 ± 3.94

Note: Data are presented as mean ± SD. Different lowercase letters within the same row indicate significant differences between groups (*p* < 0.05).

**Table 4:** The contents of enzymes related to immunity and metabolism in crayfish intestinal (*n* = 5).

Groups	SOD (U/mg)	MDA (nmol/mg)	CAT (μmoL/mg)	LZM (U/mg)	AST (nmol/mg)	ALT (nmol/mg)	LDH (nmol/mg)
Control	1.68 ± 0.34 ^b^	8.66 ± 4.26	19.97 ± 5.74	37.96 ± 8.54 ^b^	60.81 ± 9.1	73.32 ± 25.73	288.73 ± 102.83
Oregano	2.89 ± 0.77 ^a^	10.09 ± 4.12	23.09 ± 5.9	62.97 ± 16.42 ^a^	84.34 ± 23.74	94.05 ± 26.51	343.43 ± 174.6

Note: Data are presented as mean ± SD. Different lowercase letters within the same row indicate significant differences between groups (*p* < 0.05).

## Data Availability

The datasets used and/or analyzed during the current study are available from the corresponding author upon reasonable request.

## Supplementary Materials

The following supporting information can be downloaded at https://www.mdpi.com/article/10.3390/ani16182926/s1. Table S1, Amino acid content in crayfish muscle.
