# Supplementary material for: The Effects of Oregano Essential Oil on Intestinal Health and Muscle Nutritional Components of Crayfish (Procambarus clarkii)
Source: Animals (Basel). 2026 Sep 17;16(18):2926. doi: 10.3390/ani16182926 (PMC13603428; doi:10.3390/ani16182926)
Supplement: Supplementary file 1 [file animals-16-02926-s001.zip › animals-4479841-supplementary.pdf]

Supplementary Materials

Table S1. Amino acid content in crayfish muscle

Type	Control group	Treatment group
Lysine	14.02±0.93	14.73±1.11
Phenylalanine	13.32±1.56	13.27±1.43
Threonine	12.97±1.15	12.62±0.92
Valine	10.67±1.09	10.52±0.67
Methionine	15.45±2.73	13.11±1.83
Isoleucine	12.11±1.15	12.84±1.45
Leucine	22.61±1.42	26.05±4.32
Histidine	10.92±4.99	7.13±3.3
Arginine	31.16±2.71	32.18±2.09
Aspartic acid	17.76±5.55	23.16±4.95
Glutamic acid	19.01±3.85	18.14±4.04
Serine	15.45±0.66	17.49±2.57
Glycine	15.25±0.82	17.9±3.99
Alanine	15.22±1.12	16.39±3.38
Proline	6.19±0.63	5.72±0.96
Tyrosine	15.97±1.37	17.87±2.97
Cystine	4.2±0.27[b]	6.75±1.34[a]

Note: Different lowercase letters indicate significant differences between groups ($P < 0.05$).
